# Adhesion strength and anti-tumor agents regulate vinculin of breast cancer cells

**DOI:** 10.3389/fonc.2022.811508

**Published:** 2022-08-16

**Authors:** Despoina Nektaria Metsiou, Despina Deligianni, Efstathia Giannopoulou, Haralabos Kalofonos, Angelos Koutras, George Athanassiou

**Affiliations:** ^1^ Laboratory of Biomechanics and Biomedical Engineering, Department of Mechanical Engineering and Aeronautics, University of Patras, Patra, Greece; ^2^ Clinical Oncology Laboratory, Division of Oncology, Department of Medicine, University of Patras, Patra, Greece

**Keywords:** adhesion forces, elasticity, cancer cells, vinculin, anti-tumor agents

## Abstract

The onset and progression of cancer are strongly associated with the dissipation of adhesion forces between cancer cells, thus facilitating their incessant attachment and detachment from the extracellular matrix (ECM) to move toward metastasis. During this process, cancer cells undergo mechanical stresses and respond to these stresses with membrane deformation while inducing protrusions to invade the surrounding tissues. Cellular response to mechanical forces is inherently related to the reorganization of the cytoskeleton, the dissipation of cell–cell junctions, and the adhesion to the surrounding ECM. Moreover, the role of focal adhesion proteins, and particularly the role of vinculin in cell attachment and detachment during migration, is critical, indicating the tight cell–ECM junctions, which favor or inhibit the metastatic cascade. The biomechanical analysis of these sequences of events may elucidate the tumor progression and the potential of cancer cells for migration and metastasis. In this work, we focused on the evaluation of the spreading rate and the estimation of the adhesion strength between breast cancer cells and ECM prior to and post-treatment with anti-tumor agents. Specifically, different tamoxifen concentrations were used for ER^+^ breast cancer cells, while even concentrations of trastuzumab and pertuzumab were used for HER2^+^ cells. Analysis of cell stiffness indicated an increased elastic Young’s modulus post-treatment in both MCF-7 and SKBR-3 cells. The results showed that the post-treatment spreading rate was significantly decreased in both types of breast cancer, suggesting a lower metastatic potential. Additionally, treated cells required greater adhesion forces to detach from the ECM, thus preventing detachment events of cancer cells from the ECM, and therefore, the probability of cell motility, migration, and metastasis was confined. Furthermore, post-detachment and post-treatment vinculin levels were increased, indicating tighter cell–ECM junctions, hence limiting the probability of cell detachment and, therefore, cell motility and migration.

## 1 Introduction

Breast cancer is a heterogeneous disease where different metabolic phenotypes of cancer cells predispose the tumor to progression, while metastasis is one of the leading causes of death in patients with breast cancer. Hence, the investigation of biomechanical and biochemical processes and the interplay between breast cancer cell–ECM interactions that participate during metastasis is of paramount importance (1). The mechanisms of metastasis include a sequence of attachment, spreading, and detachment events, encouraged by the dissipation of cell–cell junctions and cell–ECM adhesion forces (2). Cell spreading is an essential step for the progression of cell motility, migration, and, therefore, metastasis (3). Cancer cells adhere and spread by exerting forces on the cell membrane and the ECM. Intracellular forces drive the membrane outward during spreading and stabilize cell shape in adherent and migrating cells, while the cytoskeleton plays a pivotal role in cell spreading and detachment (4). More specifically, actin polymerization and myosin contraction contribute to cell movement within the ECM in the direction of metastasis (5, 6). The process of cell adhesion is primarily achieved by connecting intracellular cytoskeletons between cells or connecting the cellular cytoskeleton with ECM components (7). The loss of cell–cell adhesion is important for developing cancer invasion and metastasis (8–10). Reduced cell–cell adhesion due to loss of E cadherin along with loss of cell–ECM local adhesion proteins may lead to epithelial-to-mesenchymal transition (EMT), a critical condition leading to the initiation of metastasis. A key adhesion-related protein regarding cell–ECM and cell–cell junctions is vinculin. New insights have established that vinculin has no enzymatic activity, while its function is still emerging (11). Specifically, vinculin regulates the transmission of contractile forces (12), which affect metastasis. The loss of vinculin is correlated with the development of many cancers, such as squamous carcinoma rhabdomyosarcoma and breast cancer.

Recent studies suggest that cell–cell adhesiveness is generally reduced in human cancers (13). Hence, reduced intercellular adhesiveness permits cancer cells to disobey the social order, contributing in dissociation of histological structure, which is the morphological hallmark of malignant tumors, thus facilitating invasion and metastasis (14). The question that arises is how would the progression of the tumor microenvironment, specifically the spreading rate and adhesion forces between untreated and treated with anti-tumor drugs, cancer cells be affected? How do these drugs affect focal adhesion formations in the ECM and therefore the metastatic cascade?

This work aimed to investigate and elucidate the metastatic potential of cancer cells by evaluating the cell stiffness, the spread and the adhesion strength of two breast cancer cell lines with different phenotypes, MCF-7/ER^+^ and SKBR-3/HER2^+^, prior to and post-treatment with the antitumor agents tamoxifen, a selective estrogen receptor modulator; and trastuzumab and pertuzumab, monoclonal antibodies against HER2. Initially, we estimated the cell stiffness *via* the micropipette aspiration technique, underlying the alterations in elastic Young’s modulus upon treatment. Furthermore, the spreading rate of the cell area prior to and post-treatment was determined. Finally, a range of shear stresses were applied to cancer cells, to detach from the ECM and therefore to evaluate the adhesion strength (15–17). More specifically, the rotating disc device was employed for estimating the cell–ECM adhesion strength of both cancer cell lines prior to and post-treatment with the aforementioned agents. To identify the role of vinculin in adhesion mechanisms, cancer cells exhibited immunofluorescence assay post-detachment event and post-treatment with anti-tumor agents. The understanding of the role of adhesion strength and spreading rate and their correlation with biochemical alterations can provide innovative insights into the process of cancer and may establish the basis for new therapeutic approaches (18–20).

## 2 Materials and methods

### 2.1 Cell culture

As previously, MCF-7 and SKBR3 (ATCC, USA) cancer cell lines were used (21). Briefly, MCF-7 was cultured in EMEM supplemented with 1 mM sodium pyruvate, 1.5 g/l sodium bicarbonate, 2 mM L-glutamine, 0.1 mM nonessential amino acids, and 0.01 mg/ml insulin (Sigma-Aldrich, Inc., USA). SKBR-3 cancer cells were cultured in DMEM supplemented with 2 mM L-glutamine. Both cell lines were supplemented with 100 μg/ml penicillin G/streptomycin, 50 μg/ml gentamycin, and 10% Fetal Bovine Serum (FBS). Phosphate Buffered Saline (PBS) was used for rinsing cells. All the above media and supplements were purchased from Biochrom (Berlin, Germany). Cells were cultured at 37°C, 5% CO_2_, and 100% humidity. Tamoxifen was purchased from Sigma-Aldrich (Sigma-Aldrich, Inc., USA) and trastuzumab (Herceptin) and pertuzumab (Perjeta) were purchased from Roche (Roche). MCF-7 cells were treated with different concentrations of tamoxifen (10, 20, and 50 nM) while SKBR-3 cells were treated with 10 μg/ml of trastuzumab or/and pertuzumab, respectively (22–24). Dexamethasone was used as a positive control at a concentration of 10^−8^ M.

### 2.2 Preparation of 2D surfaces coated with collagen I

Volume of 1 ml of collagen I (cat. no. L 7220, Biochrom) was added to a 6-well polystyrene plate with an equal volume of 1× PBS and adjusted to a pH of 3.0–3.5. The PBS solution was also adjusted to a pH of 3.0–3.5 as follows: 1 ml 1 N HCl to 100 ml PBS solution. Volume of 2 ml of the diluted collagen I solution per 10 cm^2^ area of the culture flask and incubated for 2–3 h. The solution was then removed and washed with 1× PBS (pH approx. 7.3). The 6-well plate was then immediately filled with media and cancer cells were seeded.

### 2.3 Cell elasticity

Cancer cells exhibited a micropipette aspiration technique (MA) as previously described (21). In brief, MA was used to partially aspirate the cell membrane firmly, thus avoiding nucleus aspiration, to obtain measurements for the applied negative pressure at each time point of the resulting aspirated cell elongation. A range of suction pressure DP from 0.05 to 340 Pa was applied very slowly, to achieve a linear expression of cell deformation vs. aspiration pressure to determine the elastic Young’s modulus. When the micropipette radius was exceedingly small compared to the local radius of the cell surface, the projection of cell length, *L*, into the micropipette was predicted to be proportional to the aspiration pressure Δ*P* (25). Therefore, cancer cell elasticity was determined through the slope of the curve $\Delta P=f(\frac{L}{D_{p}})$ *via* the interpreted equation: $E=\frac{R_{p}\Delta P}{2\pi L}\varphi_{p}$ (1) where E is the elastic Young’s modulus, *R_P_* the inner pipette radius, and *φ_p_* represents a function of the ratio of the pipette wall thickness to the pipette radius (*φ_p_* = 2.0–2.1 when the ratio of the pipette wall thickness to radius was equal to 0.2–1.0). Four repeated sample tests with MA were performed for each cell type condition (treated and untreated), measuring approximately 20 cells per sample.

### 2.4 Assessment of spreading rate

To address the cell spreading procedure, including untreated and treated with anti-tumor drugs tamoxifen, trastuzumab, and pertuzumab, cancer cells were seeded on a collagen I-coated 12-well plate and incubated for 12 h. The cell spread area was evaluated during incubation time of 0–12 h, and the attached cell membrane to the substrate contact area was determined by tracing the outline of the cell every hour using Sigma Scan Pro 5 software. The time-dependent normalized area was quantified by dividing the difference between the cell area at time *t*, *A_t_*, and the initial spread area *A_intial_* (the very first spread area after cell rolling upon surface) by the difference in area between the initial (*A_initial_*) and final time points (*A_final_*), where the cell was fully spread. Therefore, the cell spread area was estimated *via* the equation $A_{normalized}=\frac{A_{t}-A_{initial}}{A_{final}-A_{initial}}$ (2). The curves yielded the spreading rate of % *A_normalized_* cell area vs time for each case of control and treatment (26).

#### 2.4.1 Evaluation of isotropic and anisotropic spreading

The evaluation of isotropic or anisotropic spreading was estimated when HER2^+^ (SKBR-3) and ER+ (MCF-7) breast cancer cells were spread in the maximal area of contact with the ECM. Control SKBR-3 and MCF-7 cells were seeded on a collagen I-coated 12-well plate and incubated until the cell membrane reached its maximal spread potential. The cells were then fixed with 4% paraformaldehyde and permeabilized with 0.1% Triton X-100. Cells were rinsed with wash buffer and then blocked with bovine serum albumin in phosphate-buffered saline for 1 h at RT. After blocking, cells were labeled with conjugated Phalloidin TRITC (1:200, Merck/Millipore) for 1 h at RT and then rinsed with wash buffer. Then, Hoechst 33258 (1:4,000, Sigma-Aldrich) was used for nuclear counterstaining and then the cells were mounted. Fluorescent imaging was conducted on a Leica SP5 TCS equipped with a ×40/1.25NA oil immersion lens. Three different experiments were performed, and 100 cells were measured using Image J. Specifically, the associated aspect ratio was calculated as the minimal and maximal cell radius from the center of the nucleus in cartesian coordinates (*R_x_*,*R_y_*). An isotropic distribution of cell fibers was achieved when the aspect ratio is ˜1.

### 2.5 Assessment of cell detachment

**Figure 1:**
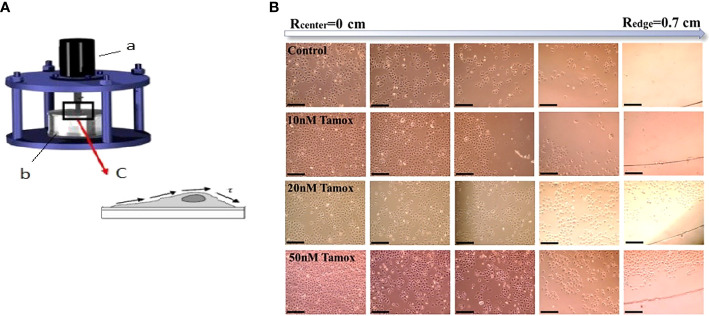
Estimation of adhesion forces by rotating disc assay. **(A)** The device consists of (a) rotor (b) tank filled with PBS at 37 °C, and (c) attached cancer cells on coverslips exhibiting shear stress (τ) (16, 17). **(B)** MCF-7 cell detachment event in different distances from the center of the disc (Rcenter = 0) to the edge (Redge = 0.7 cm) and in different tamoxifen concentrations, black scale bar 100 μm, ×10 microscope lens. Cell detachment was less favored at the highest tamoxifen concentrations.

Cells were grown in collagen coated circular glass coverslips, 0.7 cm in radius, immersed in 12-well plates filled with media and 10% FBS (40 × 10^3^ cells/well). After 24 and 48 h of incubation of MCF-7 and SKBR-3 with the relevant drugs and inhibitors, respectively. Cell adhesion forces were evaluated using a rotating disc device for each case of untreated and treated cells. This technique utilizes shear stress generated from the rotating disc upon attached to ECM cancer cells. Specifically, a rotating disc device was employed to apply shear stress to cells. The adhesion strength of the cells was determined by estimating the essential shear stress to detach the cells from their substrate (27). The rotating disc device comprises a disc clutched to the motor and a chamber made of plexiglass^®^ (27). The chamber was filled with PBS 1× solution at a constant 37 °C temperature. The aforementioned glass coverslips with the adherent cancer cells were glued to the disc and inserted into the rotation chamber, and hence, the cancer cells underwent shear stress (**Figure 1**). When the value of shear stress reaches a critical level, then 50% of the attached cancer cells are detached from the surface. The adherent fractions of cells were quantified using microscopy in combination with image processing software (Sigma Scan Pro 5). The shear stress at the surface of the rotating disc was calculated from the equation: *τ*=0.7996 *r* *ρ* *v* ^1/2^ *ω* ^3/2^ (3) where *τ* is the applied shear stress, *r* the distance to the center of the disc, *ρ* the density of the rotation buffer, *v* the kinematic buffer viscosity and *ω* the angular velocity with a range of 100–300 rpm (28). After this process, the curves of the percentage number of detached cells (referred to the distance of each cell from the center of the disc) vs required shear stress for detachment were educed and the critical shear stress for detachment was assessed. Post detachment event cells were fixed for immunofluorescence assay.

#### 2.5.1 Immunofluorescence assay after detachment

After detachment, cells were gently rinsed with PBS and fixed with 4% paraformaldehyde and permeabilized with 0.1% Triton X-100. Cells were then rinsed with wash buffer (1× PBS containing 0.05% Tween-20). Blocking was performed with 3% bovine serum albumin in phosphate-buffered saline containing 10% FBS for 1 h at RT. After blocking, cells were labeled with anti-Vinculin for 1 h. Cells were labeled with conjugated Phalloidin TRITC (1:200, Merck/Millipore) and FITC (1:200, Jackson ImmunoResearch Laboratories, Inc.) for 1 h at RT and then rinsed with wash buffer. Then Hoechst 33258 (1:4,000, Sigma-Aldrich) was used for nuclear counterstaining and then the cells were mounted. Z-stack imaging was conducted on a Leica SP5 TCS equipped with a ×40/1.25NA oil immersion lens. Overall, more than 200 cells for each case of control and treatment were studied after multiple repeated experiments (>3), and the mean fluorescence intensity of the sum projection of vinculin was evaluated after background subtraction with the use of the software ImageJ.

### 2.6 Statistical analysis

The results from cell spreading and detachment were statistically analyzed and fitted using Origin Pro9 (OriginLab Corporation, Northampton, USA) and Sigma Scan Pro 5 software for evaluating normalized cell area (A_normalized_). The results of the Elastic Young’s modulus of cancer cells were statistically analyzed using Matlab R2021a. For each case of control and treated with anti-tumor agents, 40 cancer cells were probed using a micropipette technique over three repeated experiments. Differences between the groups and controls were assessed by one-way analysis of variance (ANOVA). Each experiment included a minimum of three repeated measurements. The results were considered to be statistically significant when p <0.05.

## 3 Results

### 3.1 Elastic Young’s modulus of breast cancer cells (overview)

Breast cancer elasticity post-treatment with 10 nM tamoxifen and 10 μg/ml trastuzumab was investigated in our previous work (21). Here, we aimed to further investigate whether different concentrations of Tamoxifen or different anti-tumor agents affect cell elasticity and relate these results to the potential of breast cancer cells to spread and detach. Indeed, in **Table 1**, the Elastic Young’s modulus of both previous and current work is included. Specifically, in MCF-7 cells, we found that the Elastic Young’s modulus was markedly increased as tamoxifen concentration levels were increased. Regarding SKBR-3 cells using another anti-tumor agent, pertuzumab, the cell elasticity increased radically while the synergistic effect of trastuzumab and pertuzumab affected cell elasticity modestly regarding the isolated effect of pertuzumab.

**Table 1:** Elastic Young’s modulus of ER^+^ cancer cells in different tamoxifen concentrations and HER2^+^ cancer cells post-treatment with trastuzumab, pertuzumab, and their synergistic effect in even concentrations.

Breast cancer cells	ER^+^	HER2^+^	Drug	Concentration	Elastic modulus (Pa)		
					**Control**	**24 h of treatment**	**48 h of treatment**
MCF-7	+	−			196.3 ± 21.68 (21)		
			Tamoxifen	10 nM	–		224.64 ± 15.34^*^(21)
				20 nM		465.80 ± 30.15^*^	536.25 ± 43.42^**^
				50 nM		585.00 ± 24.80^**^	634.38 ± 35.40^**^
			Dexamethasone	10^−8^ M		375.42 ± 35.12^**^	417.03 ± 16.36^**^
					
					
SKBR-3	−	+			277.86 ± 12.57 (21)		
			Trastuzumab	10 μg/ml		–	343.62 ± 28.45^**(^ 21)
			Pertuzumab	10 μg/ml		360.89 ± 31.41^**^	559.00 ± 13.90^**^
			Trastuzumab + Pertuzumab	10 μg/ml		261.79 ± 11.80^***^	395.89 ± 45.90^***^
			Dexamethasone	10^−8^ M		186.42 ± 88.50^**^	466.45 ± 14.20^*^

Dexamethasone was used as positive control. Data express mean values ± SD of at least three repeated experiments, ^*^p <0.05, ^**^p <0.01, and ^***^p <0.001.

### 3.2 Anti-tumor agents regulate spreading rate of breast cancer cells

For estimating spreading kinetics, the experimental data of normalized cell area versus time were fitted with a sigmoidal fit, which reflects the dose–response curve. Two distinct phases of expanded cell area were observed with a different spreading rate *A_normalized_*/dt.

**Figure 2:**
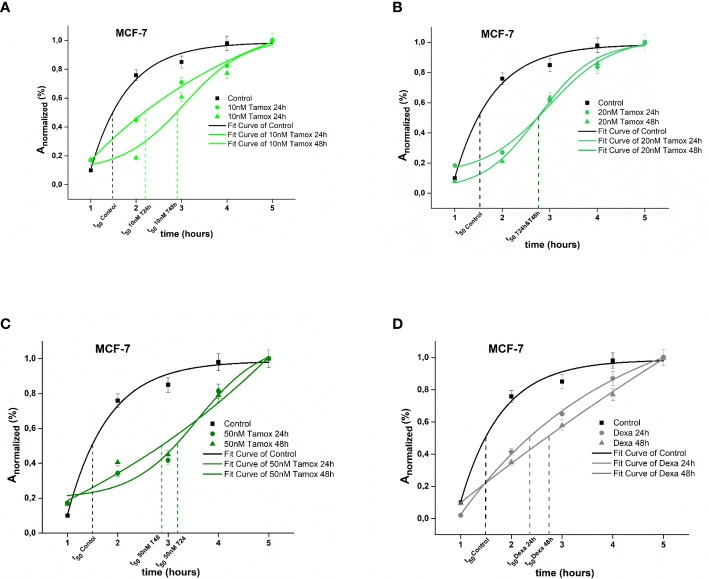
Plotted normalized cell area interprets spreading kinetics. Merged experimental curves of three repeated tests of the spreading rate (*dA_normalized_*/*dt*) of Control vs 10 nM tamoxifen **(A)**, Control vs 20 nM tamoxifen **(B)**, Control vs 50 nM tamoxifen MCF-7 **(C)** and Control vs dexamethasone **(D)** are depicted.

The spreading kinetics of MCF-7 cancer cells is depicted in **Figure 2**. Results showed that 50% of the *A_normalized_* of untreated (Control) cells was achieved in the very first 1.5 h, while the maximum spread area was integrated within 4 h (p <0.001). Regarding 10 nM of tamoxifen-treated cells, 50% of A_normalized_ was achieved within 2.17 h after 24 h of treatment, and within 2.98 after 48 h of treatment, respectively. Moreover, the maximum spread area was integrated within 4 h (p <0.001) (**Figure 2A**). Post treatment with 20 nM tamoxifen, MCF-7 cells reached the 50% of *A_normalized_* within 2.80 h in both 24 h and 48 h post-treatment (**Figure 2B**). Furthermore, 24 h and 48 h post-treatment with 50 nM tamoxifen, 50% of *A_normalized_* was achieved within 2.84 h and 3.24 h, respectively (**Figure 2C**). Positive control cells treated with dexamethasone for 24 h and 48 h reached 50% of *A_normalized_* within 2.5 and 2.65 h, respectively (**Figure 2D**). Overall, regarding MCF-7 cells, different concentrations of tamoxifen postponed the spreading event and the maximum spread cell area of treated cells was confined compared to untreated cells. MCF-7 cells were reluctantly spread post-treatment with 50 nM tamoxifen after 48 h of treatment.

**Figure 3:**
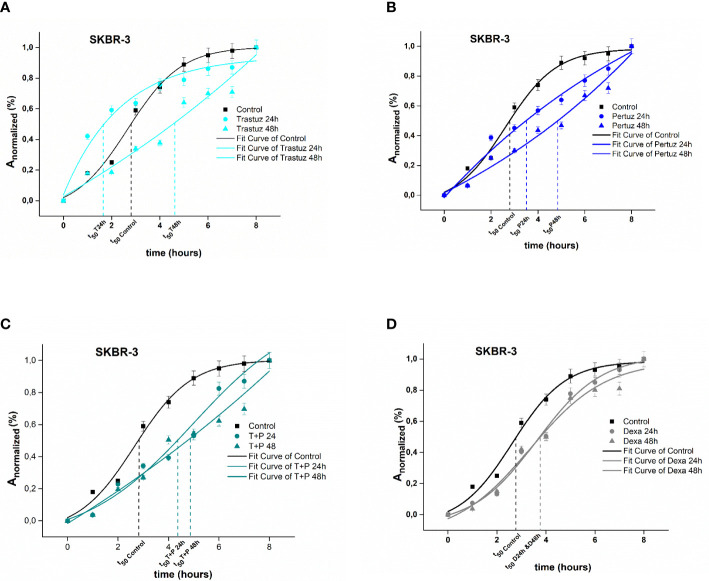
Plotted normalized cell area interprets spreading kinetics. Merged experimental curves of three repeated tests of the spreading rate (*dA_normalized_*/*dt*) of Control vs trastuzumab **(A)**, Control vs pertuzumab **(B)**, Control vs trastuzumab + pertuzumab SKBR-3 **(C),** and Control vs dexamethasone **(D)** are depicted.

In the case of SKBR-3 cancer cells, untreated (Control) cells exhibited a rapid spreading rate, where the increase of 50% of *A_normalized_* was achieved within 2.7 h following the plateau and the maximum spread area was completed within 7 h. In contrast, trastuzumab treated SKBR-3 cancer cells exhibited initially a slow spreading rate followed by a rising period of spreading rate where the increase of 50% of *A_normalized_* was integrated within 1.8 h and 4.7 h post 24 h and 48 h of treatment with trastuzumab and the maximum spread area, within 5 h (p <0.001) (**Figure 3A**). Regarding post-treatment with 24 h and 48 h of pertuzumab, SKBR-3 cells reached 50% of *A_normalized_* within 3.2 and 4.9 h, respectively (**Figure 3B**). The synergistic effect of trastuzumab and pertuzumab post 24 h and 48 h of treatment increased the time where 50% of *A_normalized_* was integrated at 4.3 and 4.8 h correspondingly (**Figure 3C**). Regarding positive control cells, 50% of *A_normalized_* achieved within 3.9 h (**Figure 3D**).

Overall, SKBR-3 cancer cells exhibit a slower spreading rate when treated with trastuzumab and pertuzumab, corroborating that in the presence of monoclonal antibodies, the attachment and the spreading rate are less favored.

### 3.3 Isotropic and anisotropic spreading

**Figure 4:**
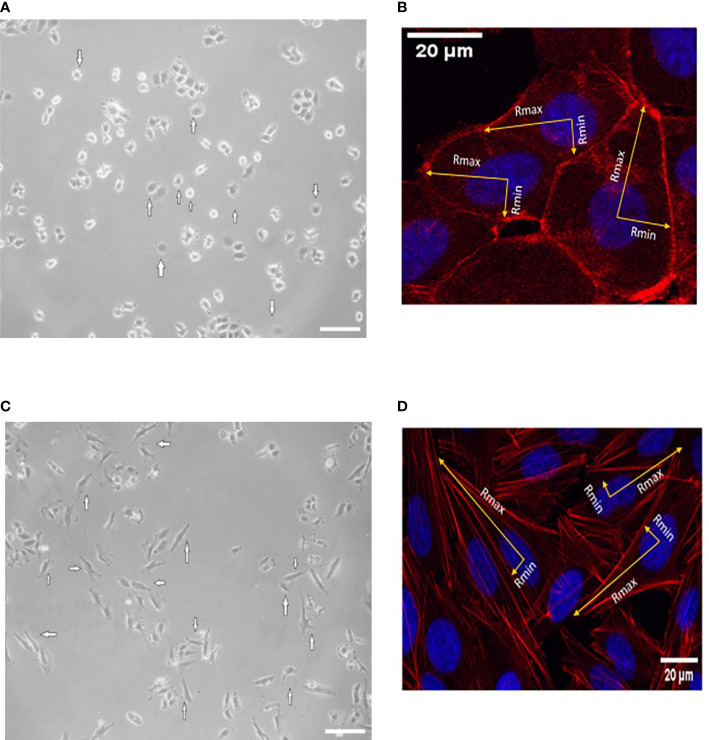
Represented images of spreading events of MCF-7 **(A, B)** and SKBR-3 **(C, D)** cancer cell lines after 20 h of incubation. White arrows indicate isotropic-like spreading in the case of MCF-7 cancer cells where the cell membrane extends and flattens evenly in all directions and a similar distance from the center of the nucleus. Anisotropic spreading occurs in the case of SKBR-3 cancer cells where the cell membrane extends unevenly from the center of the nucleus to the protrusions’ leading edge. The minimum and maximum cell radii are indicated with yellow arrows. The isotropic spread occurs when the aspect ratio of Rmax and Rmin is approximately 1. White color scale bar 40 μm, ×10 microscope lens **(A, C)** and 20 μm ×40 confocal lens (**B, D**).

To address the isotropic and anisotropic spreading, we measured the morphology of F-actin stress fibers once control MCF-7 and SKBR-3 cells were completely stressed. MCF-7 adopted a spherical like spreading with an even distribution of F-actin fibers along the cell periphery (**Figures 4A, B**). MCF-7 Rx = 21.94 ± 5.20 μm and Ry = 20.54 ± 5.12 μm with the associated aspect ratio of 1.068 (˜1). However, SKBR-3 cells presented highly elongated morphologies on dense F-actin networks (**Figures 4C, D**). The aspect ratio of SKBR-3 cells was estimated with continuous growth, implying anisotropic shape (29). Specifically, the estimated maximal and minimal radii were estimated to be Rx = 51.62 ± 11.79 μm and Ry = 6.87 ± 2.01 μm, correspondingly with the associated aspect ratio of 7.5 (>1).

### 3.4 The role of anti-tumor agents on adhesion forces of breast cancer cells

**Figure 5:**
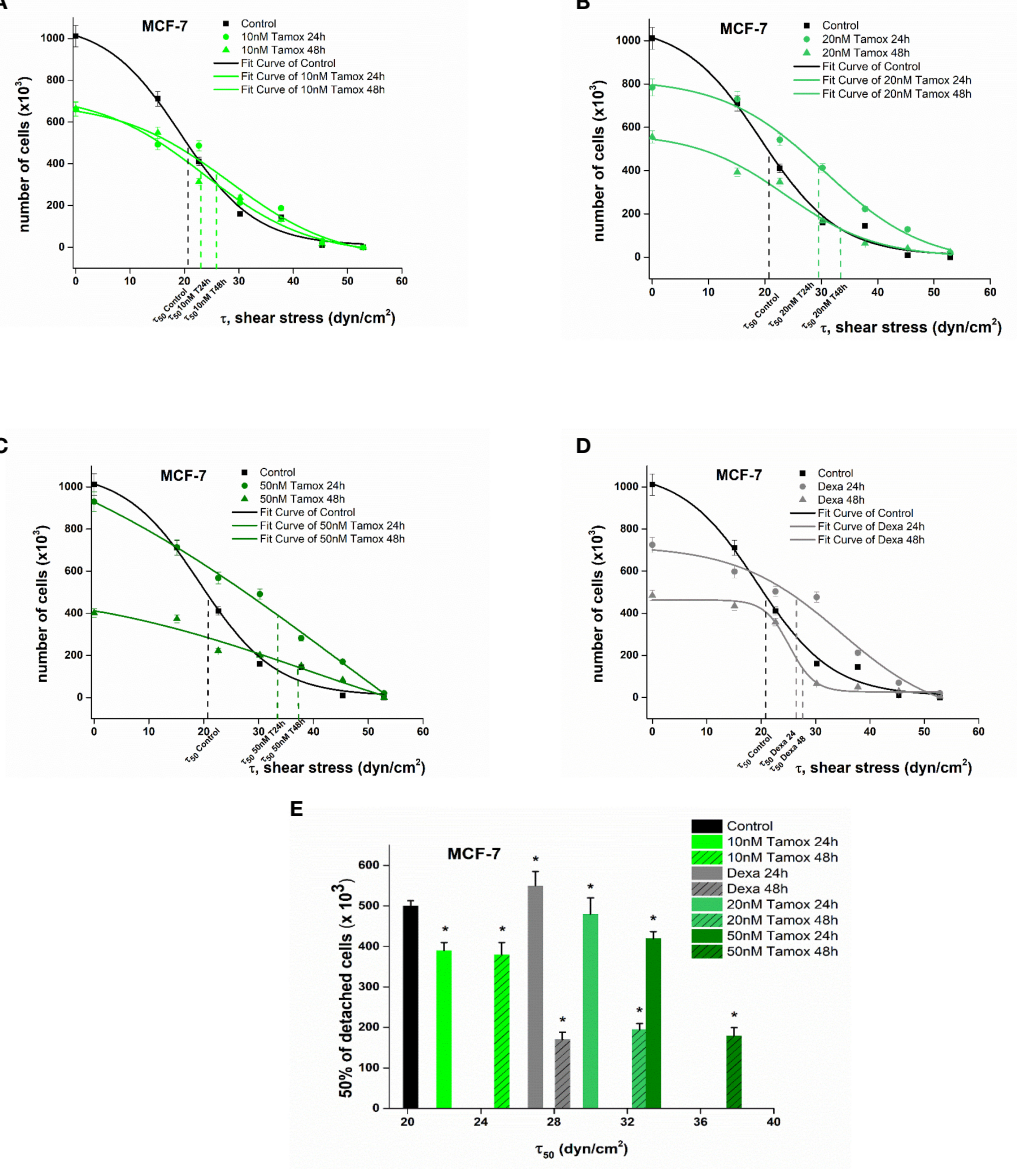
Plotted curves of the number of remaining MCF-7 cells post-detachment event vs the essential shear stress for detachment **(A–D)**. The applied shear stress is zero to attached cancer cell upon the center of the disc (r = 0) where the remained cells after detachment are 100%, no detachment was occurred and maximum when r = R_disc_ = 1 cm where all cancer cells where detached. Percentage number of remaining cells of control and treated MCF-7 breast cancer cells upon the collagen coated surface after applying a range of 0–200 dyn/cm^2^ shear stresses **(E)**. Data express mean values ± SD of at least three repeated experiments, ^*^p <0.05.

**Figure 6:**
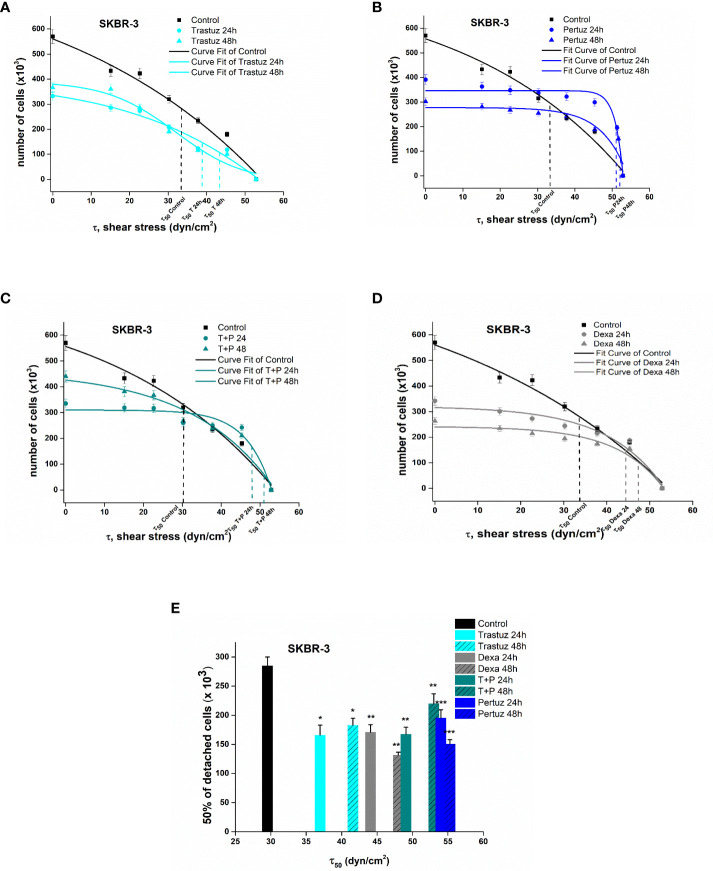
Plotted curves of the number of remaining SKBR-3 cells post detachment event vs the essential shear stress for detachment **(A–D)**. The applied shear stress is zero to attached cancer cell upon the center of the disc (r = 0) where the remained cells after detachment are 100%, no detachment was occurred and maximum when r = R_disc_ = 1 cm where all cancer cells where detached. Percentage number of remaining cells of control and treated SKBR-3 breast cancer cells upon the collagen coated surface after applying a range of 0–200 dyn/cm^2^ shear stresses **(E)**. Data express mean values ± SD of at least three repeated experiments, ^*^p <0.05, ^**^p <0.01, and ^***^p <0.001.

For the estimation of detachment forces, which is equal to the adhesion strength, a crucial detachment force was defined as the critical shear stress *τ*_50_, namely, the value of the needed force where 50% of the cells were detached from the surface of the rotating disc. **Figures 5**, **6** show the plotted curves of the number of remaining cells after the detachment event vs the essential shear stress for cells to detach from the ECM.

In the case of MCF-7 cells, untreated cancer cells revealed a critical shear stress *τ*_50_ of 21.0 dyn/cm^2^. Considering post-treatment with 10 nM tamoxifen dyn/cm^2^ (**Figure 5A**) the *τ*_50_ was at 23.5 and 25.7 dyn/cm^2^ 24 h and 48 h of post-treatment. After increasing tamoxifen to 20 nM, the relevant critical shear stress *τ*_50_was also increased to 29.8 and 33.4 dyn/cm^2^ respectively (**Figure 5B**). A further increase in tamoxifen concentration of 50 nM indicated 50% cell detachment at 34.7 and 38.5 dyn/cm^2^ (**Figure 5C**). In dexamethasone-treated cells, the critical shear stress *τ*_50_ was 27.3 and 29.2 dyn/cm^2^ after 24 h and 48 h of treatment, respectively (**Figure 5D**).

Overall, post-treatment with increased tamoxifen levels, the potential of MCF-7 to detach was decreased and the required shear stress for detachment compared to control cells was 14.2 % higher at 10 nM tamoxifen and dramatically increased at 48.5% and 54.2% at tamoxifen concentrations of 20 nM and 50 nM, 48 h post-treatment, respectively. These results imply that the increased tamoxifen concentration inhibits the detachment of treated cells and hence implies strong adhesion forces between the cell and ECM (**Figure 5E**).

In the case of SKBR-3 cancer cells, the critical shear stress *τ*_50_ for untreated cells was estimated at 32.7 dyn/cm^2^ (**Figure 5**), while in the case of trastuzumab-treated cells it was at 37.2 and 42.5 dyn/cm^2^ after 24 h and 48 h of treatment, correspondingly **(** **Figure 6A** **)**. Regarding pertuzumab-treated cells, the critical shear stress *τ*_50_ reached 54.9 dyn/cm^2^ and 55.87 dyn/cm^2^ post 24 h and 48 h of treatment, respectively **(** **Figure 6B** **)**. The synergistic effect of trastuzumab + pertuzumab revealed a prerequisite critical shear stress *τ*_50_ of 49.5 dyn/cm^2^ and 51.2 dyn/cm^2^ at 24 h and 48 h of treatment, respectively **(** **Figure 6C** **)**. Finally, critical shear stress *τ*_50_ for dexamethasone-treated cells was estimated at 44.5 dyn/cm^2^ and 48.2 dyn/cm^2^ at 24 h and 48 h post-treatment **(** **Figure 6D** **)**.

Overall, increased shear stress was essential to detach post-treated SKBR-3 cells from the ECM. Specifically, 48 h post-treatment with trastuzumab, the essential shear stress for 50% of cell detachment was 43.1% higher than control cells, while in the case of pertuzumab it was 88% higher. Moreover, in the case of both trastuzumab and pertuzumab-treated cells, the relevant shear stress *τ*_50_ was 82.5% higher. The results from the detachment study suggest that in the presence of pertuzumab, cancer cells detached reluctantly from the ECM **(** **Figure 6E** **)**.

### 3.5 Detachment forces and anti-tumor agents regulate vinculin and F-actin of breast cancer cells

**Figure 7:**
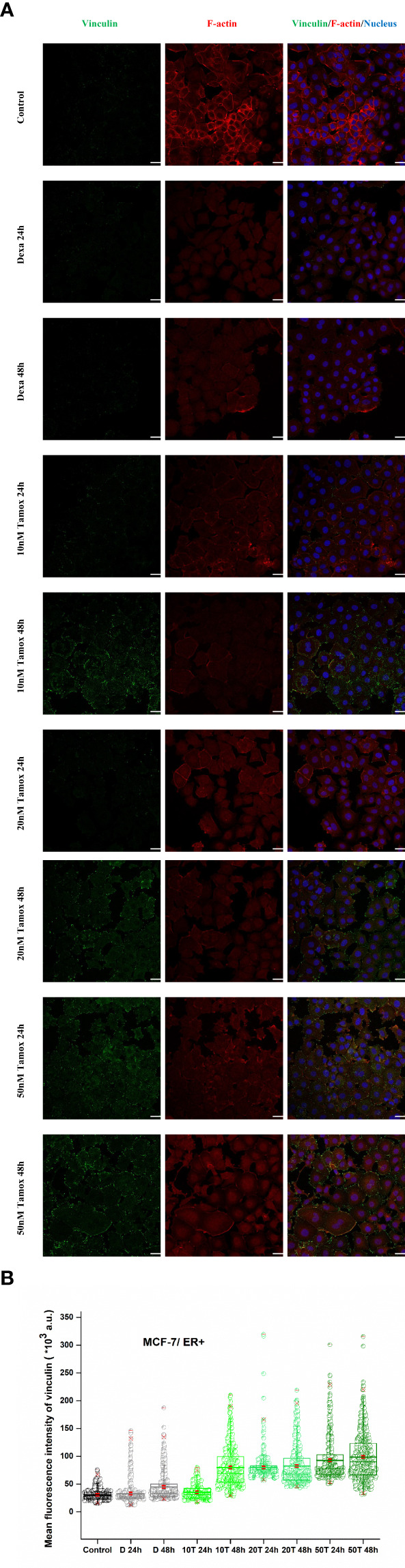
Effect of tamoxifen on vinculin expression levels. Post-detachment MCF-7 cancer cells were incubated with different concentrations of tamoxifen for 24 h and 48 h. Tamoxifen treated cells and merge plot of vinculin, F-actin and nucleus, white color scale bar 40 μm, ×40 microscope confocal lens **(A)**. Quantitative confocal microscopy analysis *via* ImageJ displays mean fluorescence intensity. Mean values ± SD of mean immunofluorescence intensity of 400 cells per treatment with three independent experiments are represented reflecting Control, decamethastone as postitive control (D 24 h, D 48 h), 10 nM tamoxifen (10 T 24 h, 10 T 48 h), 20 nM tamoxifen (20 T 24 h, 20 T 48 h) and 50 nM tamoxifen (50 T 24 h, 50 T 48 h). Box-plot whiskers represent the 5th and 95th percentile, the median value is represented by the line within the box, and the red square box depicts the mean **(B)**.

In the case of MCF-7 cells, vinculin expression levels were markedly increased post-treatment with tamoxifen. Initially, vinculin levels of control, positive control (Dexa 24 h, 48 h), and post-treated with 10 nM tamoxifen for 24 h were at approximately even fluorescence intensities as depicted in **Figures 7A, B**. However, as the concentration of the drug increases, the vinculin mean intensity also increases. Notably, after 48 h of treatment with 10 nM, 20 nM, and 50 nM tamoxifen, vinculin showed a redistribution and increased signal levels (**Figures 7A, B**), indicating a post-treatment vinculin upregulation. Moreover, the morphology of F-actin cytoskeleton protein was not altered post-treatment. However, high levels of F-actin were detected in control cells following a reduced expression in treated cells. These results corroborate the findings from the Elastic Young’s modulus evaluation, as increased post-treatment cell stiffness indicates confined migration potential (21).

**Figure 8:**
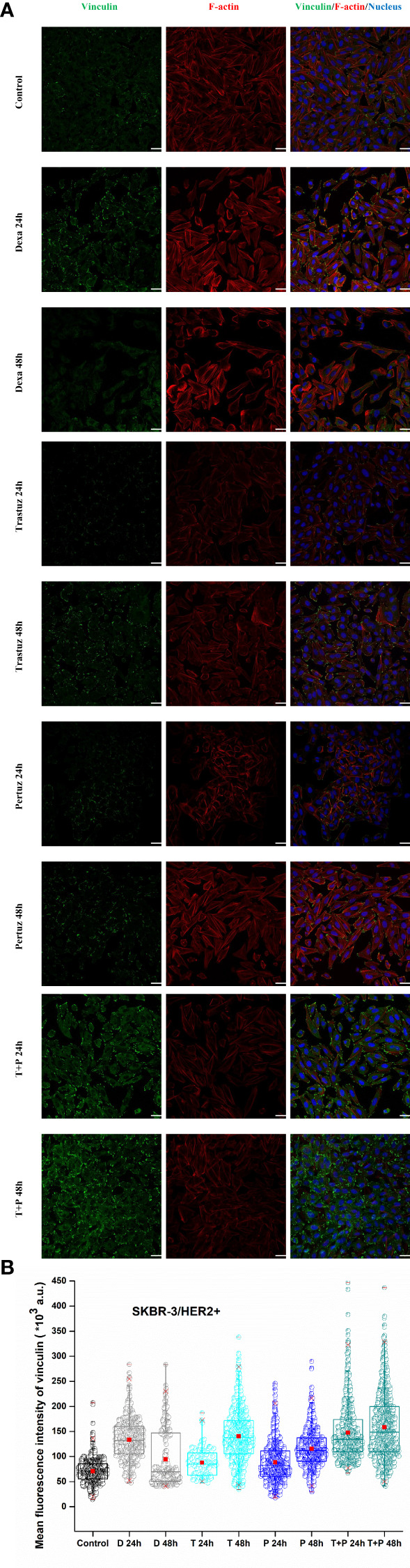
Effect of trastuzumab, pertuzumab, and their synergistic effect for double inhibition of HER2^+^ cancer cells on vinculin expression levels and merge plot of vinculin, F-actin, and nucleus, white color scale bar 40 μm, ×40 microscope confocal lens **(A)**. After detachment, SKBR-3 cancer cells were incubated with monoclonal antibodies, trastuzumab and/or pertuzumab for 24 h and 48 h. Quantitative confocal microscopy analysis *via* ImageJ displays the mean fluorescence intensity of vinculin. Mean values ± SD of mean immunofluorescence intensity of 400 cells per treatment with three independent experiments are represented reflecting Control, dexamethasone as positive control (D 24 h, D 48 h), trastuzumab (T 24 h, T 48 h), pertuzumab (P 24 h, P 48 h), and their synergistic effect (T + P 24 h, T + P 48 h). Box-plot whiskers represent the 5th and 95th percentile, the median value is represented by the line within the box, and the red square box depicts the mean **(B)**.

Regarding SKBR-3 cells, vinculin mean fluorescence intensity was significantly increased 48 h post-treatment with trastuzumab, pertuzumab, and trastuzumab + pertuzumab, as depicted in **Figures 8A, B**. Furthermore, the distribution of F-actin stress fibers was altered post-treatment. Fully polymerized F-actin in control SKBR-3 cells with extended lamellipodia and filopodia indicates a high density of F-actin cross-linking, which, according to previous studies, is related to soft and therefore more elastic cells with greater potential for migration. However, in the case of post-treated SKBR-3 cells, F-actin stress fibers were depolymerized and perinuclear accumulated, revealing a roundish cell with confined protrusions. The results of increased vinculin levels with limited cell protrusions conform to the increased cell stiffness post-treatment with anti-tumor agents, indicating confined cell motility.

## 4 Discussion

In the series of steps that comprise the metastatic potential of cancer cells, the spreading rate upon ECM and the adhesion forces between cell and ECM are inextricably linked with the progression of the disease (4, 30, 31). Biological and biochemical mechanisms of cancer cells alter and remodel the structure of the cytoskeleton to invade the ECM. Altered cytoskeleton proteins result in changes in the ability of cancer cells to contract, spread, and move upon ECM, thus influencing their biomechanics (32). As pathological tumor progression leads to aberrant biomechanical behavior in cells and tissues, thereby affecting the tumor microenvironment and therefore participating in the malfunction of cell–ECM homeostatic equilibrium (33). Nevertheless, the influence of adhesion forces between treated with anti-tumor agent cancer cell–ECM junctions and the spreading rate of treated cancer cell membranes has not been thoroughly elucidated. In this work, we compared two breast cancer cell lines with different profiles regarding their aggressiveness, to investigate the treatment mechanisms in Elastic Young’s modulus, spreading rate, adhesion forces, and focal adhesion protein vinculin levels. These parameters reflect the biological and biochemical interactions between cells and the ECM, by expressing macroscopically cell biomechanics and hence indicating the mechanical behavior of malignant cells (34). Consequently, the biomechanical properties of cancer cells could be used as indicators of their biological state or metastatic potential.

Several studies have established that the high stiffness of the ECM in the tumor microenvironment contributes to cancer progression and triggers malignant transformation (35–37). Clinical studies suggest that the collagenous matrix stiffens the tumor stroma, which directly increases tumor cell proliferation and enhances metastatic colonization, and growth (38, 39). Here, we were intrigued to extend our previous work and test the hypothesis of whether different drug concentrations affect the Elastic Young’s modulus in the case of MCF-7 cells and whether different anti-tumor agents and their synergistic effects could regulate the cancer cell elasticity of SKBR-3 cancer cells. Indeed, we estimated that in the case of MCF-7 cells, the Elastic Young’s modulus increased as tamoxifen levels were increased from 10 to 50 nM and significantly after 48 h of treatment. Regarding SKBR-3 cancer cells, we conclude that the cell stiffness increased markedly in post-treated cells with monoclonal antibodies. Interestingly, in the presence of pertuzumab, the Elastic Young’s modulus increased in a greater rate compared with trastuzumab or their synergistic effect. Overall, the increased post-treatment cell stiffness in both ER^+^ and HER2^+^ cancer cells indicates a less deformable cytoskeleton, resulting in a confined potential for cell movement (**Table 1**) (21, 40).

Regarding spreading rate, our results suggest that in both cancer cell lines, after treatment, there was no enhancement of the spreading rate, which reflects a confined extension of the cell membrane in both MCF-7 and SKBR-3 cancer cells. Hypothesizing that spreading rate reflects the metastatic potential, the finding of the same post treatment spreading rate in both breast cancer cell lines, implies an equal metastatic potential indicating the endogenous feature of aggressive phenotype of cancer cells (**Figures 2**, **3**). Comparing the two different cancer cell lines of this study, SKBR-3 and MCF-7, we showed that untreated SKBR-3 cells, as HER2^+^ belongs to highly metastatic breast cancer cells with an aggressive metastatic phenotype, and therefore the slope of the curve of the spreading experiment increased more rapidly than MCF-7 cells. In contrast, the results of untreated MCF-7 cancer cells are in line with recent studies which showed that MCF-7 cells are weak metastatic cells with less invasive potential, and therefore the spreading rate procrastinates a lot compared to SKBR-3 cancer cells (41). Interestingly, regarding post-treatment results, the spreading rate of treated SKBR-3 cancer cells indicates an expeditious progress of the phenomenon, compared to tamoxifen-treated MCF-7 cancer cells. This is in line with clinical evidence that ER^+^ breast tumors are less aggressive than HER-2^+^ tumors (42). Previous studies established that spreading could occur either isotropically or anisotropically (43, 44). Isotropic spreading occurs when the cell membrane flattens equally in all directions. On the contrary, anisotropic spreading occurs when cells produce increased membrane extensions or pseudopod protrusions. In this work, MCF-7 cancer cells followed an isotropic spread (**Figures 4A, B**) while SKBR-3 showed an anisotropic spread (**Figures 4C, D**), indicating that SKBR-3 cancer cells present a more aggressive profile with increased protrusions compared to MCF-7. Furthermore, this might imply that SKBR-3 cells might be polarized, and membrane receptors related to movement are not allocated all over the membrane of cells (45).

Cellular forces are primarily generated in the cytoskeleton, which is responsible for maintaining the cell shape and organization, imparting specific mechanical properties to cells (46). In malignant transformation, the reorganization of the cytoskeleton occurs with a simultaneous loss of strong intracellular forces, contributing to a softer cancer cell with the ability to migrate easily upon ECM (47). Additionally, according to several studies, the cell–cell adhesiveness of malignant cells is generally reduced (48, 49). Hence, cancer cells readily degrade the ECM and surrounding tissues and converge to a highly aggressive and metastatic phenotype. Regarding the results of the detachment experiment with the rotating disc and correlating treated and untreated cells, we established that the adhesion strength in case of treated cancer cells was found to be 50% higher in MCF-7 and 25% higher in SKBR-3 cells (**Figures 5**, **6**) (**Table 2**). These findings suggest that the external mechanical stimuli *via* shear stress induced biological pathways in post-treated cells which triggered the overexpression of focal adhesion vinculin in both intercellularly and cell–ECM junctions, thus impeding cancer cells from detaching from the ECM and hence confining the motility, migration, and metastatic potential (50, 51).

**Table 2:** Cumulative results of MCF-7 and detachment of SKBR-3 breast cancer cells upon treatment.

Breast cancer cells	ER	HER2	Drug	Concentration	Control		24 h		48 h	
					$\tau_{50}\left(\frac{dyn}{cm^{2}}\right)$	N_50_ (×10^2^)	$\tau_{50}\left(\frac{dyn}{cm^{2}}\right)$	N_50_ (×10^2^)	$\tau_{50}\left(\frac{dyn}{cm^{2}}\right)$	N_50_ (×10^2^)
MCF-7	+	−			21.00	505				
			Tamoxifen	10 nM			23.50	330.00	25.70	332.00
				20 nM			29.80	392.00	33.40	278.00
				50 nM			34.70	465.00	38.50	201.00
			Dexamethasone	10^−8^ M			27.30	362.00	29.20	242.00
SKBR-3	−	+			32.70	285				
			Trastuzumab	10 μg/ml			37.20	166.00	42.50	183.00
			Pertuzumab	10 μg/ml			54.90	195.00	55.87	151.00
			Trastuzumab + Pertuzumab	10 μg/ml			49.50	167.00	51.20	220.00
			Dexamethasone	10^−8^ M			44.50	171.00	48.20	131.00

τ_50_ in $\left(\frac{dyn}{cm^{2}}\right)$ is the essential shear stress to detach 50% of the cell population and N_50_ is the relevant number of detached cells.

In the existing literature, the loss of vinculin is linked to the development of many cancers (10, 52, 53). Our study conforms to other studies, which showed that overexpression of vinculin causes reduced cell migration, whereas knockdown of vinculin-enhanced cell motility (54, 55). Moreover, the study by Toma-Jonik et al. showed an association between the downregulation of vinculin, the reduced adhesion and the enhanced motility of cells over-expressing active heat shock transcription factor 1 (HSF1), the major regulator of stress response, which is frequently activated in cancer (56). Coll et al. inactivated the vinculin gene in mouse embryonal carcinoma cell lines and embryonic stem cells, and their results showed that the loss of vinculin resulted in increased cell motility (57). Sadano et al. showed that highly metastatic melanoma cells lacked vinculin or expressed only scant amounts (58). Other studies have estimated that vinculin overexpression leads to stronger adhesion and less motility (12, 54). Therefore, our data are in line with these studies, as we showed that high post-treatment vinculin expression, in both ER^+^ and HER2^+^ cancer cells (**Figures 7A, B**, **8A, B**), correlates with decreased potential for detachment and, therefore, migration upon ECM from the yielded evidence of detachment, spreading and cell stiffness. However, *in vivo* studies must elucidate and decipher the focal adhesion distribution and localization regarding post-treatment with anti-tumor agents in cancer cells to establish the correlation between knockout of vinculin and restricted cell motility.

This study aimed to understand the regulation of cell adhesion dynamics and the role of cell adhesion protein vinculin of breast cancer cells prior to and post-treatment with anti-tumor agents and their correlation to cell motility by identifying the cell stiffness and the potential for cell spread. Moreover, cell detachment was markedly confined post-treatment with various levels of tamoxifen concentration in the case of MCF-7 cells and post-treatment with trastuzumab, pertuzumab, and their synergistic effect, in SKBR-3 cells. Our results suggest a limited potential for cell movement post-treatment with the aforementioned anti-tumor agents in both breast cancer cells, and this hypothesis was enhanced by elevated mean fluorescence intensity and, therefore, overexpression of vinculin in post-treated cells. This study of MCF-7 and SKBR-3 breast cancer, which reflects the patients with ER^+^ and HER2^+^ breast cancer, respectively, and may contribute and provide new insights into new therapeutics that are about to influence both the biochemical and biomechanical responses of cancer cells.

## Data availability statement

The raw data supporting the conclusions of this article will be made available by the authors, without undue reservation.

## Author contributions

DM: Study, conception, design, performed the experiments, and data analysis. DD: Supervising and review. EG: Supervising and review. HK: Supervising and review. AK: Supervising, review, study, and conception. GA: Supervision, Review, Study, and Conception. All authors contributed to the article and approved the submitted version.

## Funding

This research was supported by funding from the source: “Constantin Carathéodory, E.044, 2013”.

## Acknowledgments

The authors want to thank the members of the Laboratory of General Biology, School of Medicine, University of Patras, for providing the Leica confocal microscopy.

## Conflict of interest

The authors declare that the research was conducted in the absence of any commercial or financial relationships that could be construed as a potential conflict of interest.

## Publisher’s note

All claims expressed in this article are solely those of the authors and do not necessarily represent those of their affiliated organizations, or those of the publisher, the editors and the reviewers. Any product that may be evaluated in this article, or claim that may be made by its manufacturer, is not guaranteed or endorsed by the publisher.
